# Detection and identification of NAP-2 as a biomarker in hepatitis B-related hepatocellular carcinoma by proteomic approach

**DOI:** 10.1186/1477-5956-6-10

**Published:** 2008-03-10

**Authors:** Min He, Jian Qin, Rihong Zhai, Xiao Wei, Qi Wang, Minhua Rong, Zhihua Jiang, Yuanjiao Huang, Zhiyong Zhang

**Affiliations:** 1 Medical Scientific Research Center, Guangxi Medical University, Nanning, 530021, P. R. China; 2 School of Public Health, Guangxi Medical University, Nanning, 530021, P. R. China; 3 Department of Environmental Health, Harvard School of Public Health, 665 Huntington Avenue, Boston, MA 02115, USA

## Abstract

**Background:**

**A **lack of sensitive and specific biomarkers is a major reason for the high rate of Primary hepatocellular carcinoma (HCC)-related mortality. The aim of this study was to investigate potential proteomic biomarkers specific for HCC.

**Methods:**

81 patients with hepatitis B-related HCC and 33 healthy controls were randomly divided into a training set (33 HCC, 33 controls) and a testing set (48 HCC, 33 controls). Serum proteomic profiles were measured using Surface-enhanced laser desorption/ionization-time-of-flight mass spectroscopy (SELDI-TOF-MS).) A classification tree was established by Biomarker Pattern Software (BPS). Candidate SELDI peaks were isolated by tricine-SDS-PAGE, identified by HPLC-MS/MS and validated by immunohistochemistry (IHC) in liver tissues.

**Results:**

A total of 6 proteomic peaks (3157.33 m/z, 4177.02 m/z, 4284.79 m/z, 4300.80 m/z, 7789.87 m/z, and 7984.14 m/z) were chosen by BPS to establish a classification tree with the highest discriminatory power in the training set. The sensitivity and specificity of this classification tree were 95.92%, and 100% respectively in the testing set. A candidate marker of about 7984 m/z was isolated and identified as neutrophil-activating peptide 2 (NAP-2). IHC staining showed that NAP-2 signals were positive in HCC tissues but negative in adjacent tissues.

**Conclusion:**

The NAP-2 may be a specific proteomic biomarker of hepatitis B-related HCC.

## 1. Background

Primary hepatocellular carcinoma (HCC) is one of the most lethal malignancies worldwide. It is the third leading cause of cancer death in China and the sixth most common cancer in the world [1,2]. Prognosis of HCC remains poor, mainly due to the failure of early diagnosis of the disease in symptom-free patients [3,4]. In contrast, early detection of HCC before the onset of clinical symptoms can lead to curative treatment, significantly improving prognosis [5,6].

At present, alpha-fetoprotein (AFP) and des-γ-carboxy prothrombin (DCP) are the two most widely used tests to aid in the diagnosis and monitoring for HCC. However, AFP is found to be normal in around one third of patients with small (<3 cm) HCC [7], and the specificity of AFP is only about 68.2% in detecting the patients with HCC [8]. Although DCP was believed to be a better marker for diagnosis of HCC, elevated DCP activity is only present in 44–47% of HCCs less than 3 cm in size [9,10]. Thus, both AFT and DCP are not ideal biomarkers for early diagnosis of HCC. There is a pressing need to find new biomarkers for more effective detection of HCC.

Recent advances in proteomic analysis have offered exciting opportunities for finding novel biomarkers in biological fluids. Today, two major strategies are used to discover clinically useful biomarkers from a proteomic approach. One of them utilizes a SELDI-TOF system [11-14], and the other is a method based on 2-D PAGE coupled with MS [15,16]. The SELDI-TOF, which generates the protein patterns by MS, has been considered a powerful tool for the discovery of new biomarkers [16,17]. However, almost all of the proteins' peaks detected by SELDI are not easily identified as protein molecules. Thus, these findings cannot provide any information about the biological roles of the marked proteins in the pathogenesis of a disease. On the other hand, the 2-D PAGE-based method provides a lot of information about proteins, including expression volumes, actual p/s and molecular weights [18]. However, the 2-D method is not sensitive for resolving hydrophobic, low abundant, low molecular weight proteins. LC-MS/MS is a powerful identification tool for proteins, but it is not suitable for analyzing proteins directly [18]. It has been suggested that the combination of SELDI-TOF, 2-D and LC-MS/MS may provide a better solution to identify disease-associated proteomic biomarkers [18,16,19].

The aim of this study was to identify serum protein biomarkers in HCC. The analysis was performed using SELDI-TOF-MS technology to screen potential protein patterns specific for HCC. Candidate protein peaks were then separated by Tricine-SDS- PAGE and trypsin digestion, identified by HPLC-MS/MS analysis and database search, and confirmed by immunohistochemistry (IHC) in liver tissues.

## 2. Results

### 2.1 Quality control of SELDI analysis

The reproducibility of SELDI spectra, *i.e*., mass location and intensity from array to array on a single chip (intra-assay) and between chips (inter-assay), was determined using the pooled normal serum quality control samples. Seven protein peaks in the range of 3,000–10,000 m/z on the observed spectra were randomly selected to calculate the coefficient of variance. The intra-assay and inter-assay coefficient of variance for peak location was 0.05%, and the intra-assay and inter-assay coefficients of variance for normalized intensity (peak height or relative concentration) were 6% and 14% respectively (data not shown). Masses that were within 0.08% mass accuracy between spectra were considered to be the same. Most importantly, it was observed that randomly selected samples, blinded to the person performing SELDI and rerun months or even a year later, were correctly classified by the decision tree classification algorithm.

### 2.2 Protein peak detection and data preprocessing

**Figure 1:**
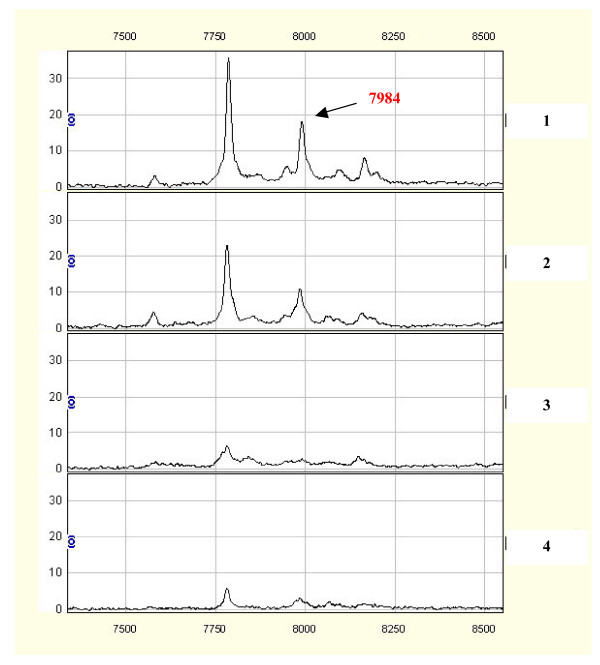
Comparisons of differential expressions of the SELDI peaks at 7984 m/z in HCC (1, 2) and healthy controls (3, 4).

Initially, we analyzed serum samples from the training set, using the Ciphergen Biomarker pattern software. Among 126 qualified mass peaks (signal-to-noise ratio>5; ranging from 2 to 50 km/z), 21 protein peaks were over-expressed, whereas 44 protein peaks were significantly downregulated in sera from HCC patients compared with those from controls (all p values<0.05; Table 1). Fig. 1 shows an example of the 7984 m/z peak that was up-regulated in HCC samples vs. control samples.

**Figure 2:**
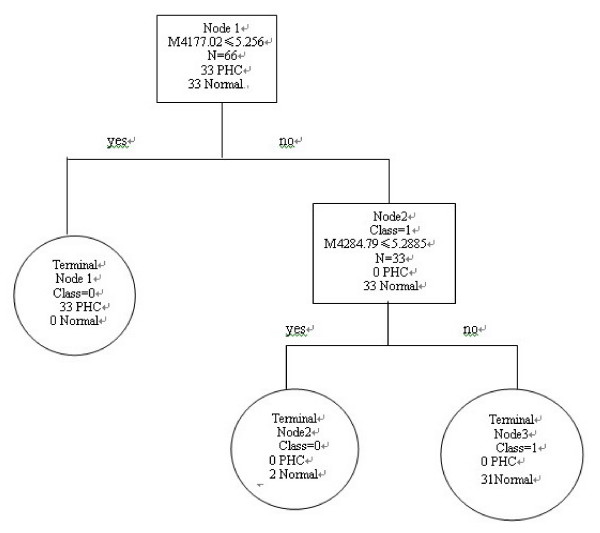
**Diagram of the classification tree for patients with HCC and healthy controls**. The squares are the primary nodes and the circles indicate terminal nodes. The mass value in the root nodes is followed by the intensity value. For example, the question forming the first splitting rule is: Are the intensity levels of the peak at 4177.02 m/z lower or equal to 5.256? Samples that follow the rule go to the left "yes" terminal node, and samples that do not follow the rule go to a "no" daughter node to the right. The number of control or HCC samples in each node are shown.

To identify biomarkers with the potential to detect HCC, the intensities of protein peaks in training set were transferred to BPS. A total of 6 peaks with the highest discriminatory power (3157.33 m/z, 4177.02 m/z, 4284.79 m/z, 4300.80 m/z, 7789.87 m/z, and 7984.14 m/z) were automatically selected to construct a classification tree (Table 2, 3). Fig. 2 shows the tree structure and sample distribution. This classification model distinguished serum samples based on their profiles of the protein peak intensities. The classification tree using the combination of these 6 protein peaks identified 33 cases of HCC and 33 controls, resulting in a sensitivity of 100% and a specificity of 96.97% respectively. (Table 4)

**Table 1:** Intensities of protein peaks between patients with HCC and controls

M/Z	*p *value	HCC patients		Controls		Expression of biomarkers
			
		Mean	SD	Mean	SD	
4177.021	0	2.4109626	1.7831004	17.790127	12.3304	↓
4284.793	1E-10	4.1675763	2.8210263	18.165137	10.96188	↓
3157.338	2E-10	2.8711763	2.3671242	16.269795	12.3949	↓
2210.294	4E-10	1.2477808	1.4009605	4.5723959	1.643278	↓
3976.459	8E-10	2.6239766	3.4075661	10.778571	7.532834	↓
2931.897	7.6E-09	3.1186882	2.0358896	11.507879	7.729	↓
3954.075	1.11E-08	1.6472851	1.2559403	7.6936686	5.96494	↓
7789.876	3.13E-08	21.662616	9.007951	8.8290885	6.779427	↑
3190.271	3.9E-08	3.5195939	3.2358447	12.027547	8.698558	↓
2880.784	7.45E-08	1.102611	1.3353521	5.001807	4.339702	↓
2042.81	8.59E-08	5.2251662	4.7882565	15.394446	8.244651	↓
4300.805	1.14E-07	6.3052246	2.8188831	14.24708	6.619937	↓
2684.091	3.21E-07	3.262482	3.6567613	9.551285	5.764105	↓
9312.108	3.21E-07	15.704431	4.4922866	8.8978734	4.287706	↑
3272.949	8.18E-07	1.8316758	1.3430214	6.5194098	5.123064	↓
8168.558	2.15E-06	4.2784744	1.7546221	2.2906521	1.328373	↑
7984.144	1.12E-05	6.0715178	4.4504467	2.9324679	1.787027	↑
2233.538	2.99E-05	1.9147055	1.5778554	4.0521515	2.028355	↓
2188.991	3.54E-05	2.5010757	1.5294086	4.3043581	1.881263	↓
2135.285	4.42E-05	3.6300916	3.49041	10.966854	9.955057	↓
11713.42	4.67E-05	4.1459834	5.4995663	1.336294	3.257604	↑
2053.145	5.5E-05	9.7060752	5.9603503	4.3023177	2.231138	↑
11803.06	5.5E-05	1.6426482	1.3296893	0.7800243	0.476692	↑
11913.52	6.47E-05	1.3968035	1.0639529	0.6868796	0.606584	↑
7577.259	8.47E-05	6.5419175	6.7324834	2.9342635	2.346913	↑
3328.797	9.93E-05	4.2466182	2.9569092	6.2898423	2.27614	↓
2458.656	0.00011	5.1397003	9.6317988	12.275455	14.48544	↓
2175.988	0.000116	2.7234278	1.2771298	4.1339189	2.387265	↓
2760.934	0.000338	4.6648972	3.2035945	9.3423629	6.483809	↓
2226.912	0.000452	2.355006	1.6168492	3.8420929	1.696412	↓
3042.037	0.000575	2.0878758	1.5066385	3.9896521	2.661699	↓
3315.627	0.000602	9.5689729	4.8592784	15.151073	7.432231	↓
2025.722	0.000694	4.6839614	3.8381819	1.7500306	1.539545	↑
2798.165	0.000694	2.0897574	2.4307372	3.9262301	3.225462	↓
3142.417	0.000875	3.1393877	1.8539336	6.0137931	4.371349	↓
3497.766	0.001004	1.9353972	2.3046427	4.1676774	3.520588	↑
2604.949	0.001258	1.6948706	2.4206924	3.5161364	2.838326	↓
2635.516	0.001316	1.8316855	2.6597455	3.412366	2.418701	↓
1986.694	0.001503	3.9645947	2.1700129	6.1885737	2.961989	↓
11546.56	0.001503	2.247573	3.0491879	0.6758139	1.142265	↑
5811.636	0.00204	6.4141252	7.5516614	2.3012091	1.338683	↑
10292.36	0.002223	1.8987997	1.2501286	1.0377238	0.735765	↑

4118.351	0.003676	7.11073	5.1441046	11.680701	6.853008	↓
3264.446	0.00668	6.9932534	5.2366332	11.0494	7.096711	↓
2149.367	0.006943	3.7005272	3.7552454	5.9585474	5.335906	↓
2011.105	0.007497	2.6144135	2.0116598	4.2731103	2.674531	↓
2785.17	0.007497	2.5972155	2.4631704	4.4992097	3.047729	↓
3472.829	0.00906	3.1882635	3.4294237	4.5476185	2.91472	↓
5482.928	0.011735	4.8059806	2.9804352	3.4096367	2.533875	↑
3923.203	0.01217	2.4220346	2.3048585	4.3790796	3.64184	↓
2316.308	0.017976	3.4762243	3.7179706	1.6393136	1.780269	↑
6845.999	0.01926	4.9948325	2.3817092	6.838496	3.161917	↓
2692.421	0.019931	4.921541	2.7549713	3.457848	2.217777	↑
3104.495	0.019931	1.3934316	1.4045028	2.3921117	1.843127	↓
2753.121	0.021336	4.4069341	3.9525245	6.3553599	4.472233	↓
3509.35	0.02207	4.5393277	3.3610221	7.2480107	5.319909	↓
6203.673	0.02207	3.3648117	1.6676335	4.5994989	2.159071	↓
9212.971	0.024405	43.945799	32.384871	24.707158	29.86224	↑
4137.329	0.026951	5.4829547	2.9809693	7.0825919	2.943026	↓
9421.615	0.032739	8.5502217	5.7753764	6.0635598	6.172742	↑
5318.755	0.037162	5.2174103	6.0996661	10.364681	11.18352	↓
2953.161	0.039559	9.7117587	7.368618	12.899569	8.162569	↓
4599.027	0.040805	18.449161	16.04898	13.034537	17.97882	↑
7988.252	0.042085	6.4870797	3.9637198	5.3343441	4.792026	↑
4858.969	0.049004	4.0595446	4.2862607	5.63334	4.377429	↓

↑:Protein peak intensity that was upregulated in the serum of HCC patient.

↓ : Protein peak intensity that was downregulated in the serum of HCC patient

**Table 2:** The intensities of six protein peaks of the classification tree in patients and controls

M/Z	*p *value	HCC patients		Controls		Expression of biomarkers
			
		Mean	SD	Mean	SD	
3157.338	2E-10	2.871176	2.367124	16.26979	12.3949	↓
4177.021	0	2.410963	1.7831	17.79013	12.3304	↓
4284.793	1E-10	4.167576	2.821026	18.16514	10.96188	↓
4300.805	1.14E-07	6.305225	2.818883	14.24708	6.619937	↓
7789.876	3.13E-08	21.66262	9.007951	8.829089	6.779427	↑
7984.144	1.12E-05	6.071518	4.450447	2.932468	1.787027	↑
11803.06	5.5E-05	1.642648	1.329689	0.780024	0.476692	↑

↑:Protein peak intensity that was upregulated in the serum of HCC patient.

↓ : Protein peak intensity that was downregulated in the serum of HCC patient

**Table 3:** Important peaks selected by BPS

M/z of candidate peaks	Score	
M4177_02	100.00	||||||||||||||||||||||||||||||||||||||||||
M4284_79	89.84	||||||||||||||||||||||||||||||||||||||
M3157_33	76.56	||||||||||||||||||||||||||||||||
M7789_87	75.62	||||||||||||||||||||||||||||||||
M4300_80	42.93	|||||||||||||||||
M7984_14	38.65	||||||||||||||||

**Table 4:** Performance of the classification tree in the training and testing sets

Sets	Groups	Sensitivity	Specificity
Training set	33cases/33 controls	100% (33/33)	93.94% (31/33)
Testing set	48cases/33 controls	100% (48/48)	96.97% (32/33)

### 2.3 Validation of the serum proteomic profiles in testing set

To evaluate the accuracy and validity of the classification tree generated from the training set, we determined the performance of the classification model in our testing dataset, consisting of 48 HCC and 33 of control samples. Consistent with the results in training set, this classification tree separated HCC samples from control samples with a sensitivity of 100%, a specificity of 96.97%, and a positive predictive value of 95.92%, respectively (Table 4). The area under the receiver operating characteristics (ROC) curve of this model was 0.986, indicating possible diagnostic utility.

### 2.4 Biomarker purification and identification

**Figure 3:**
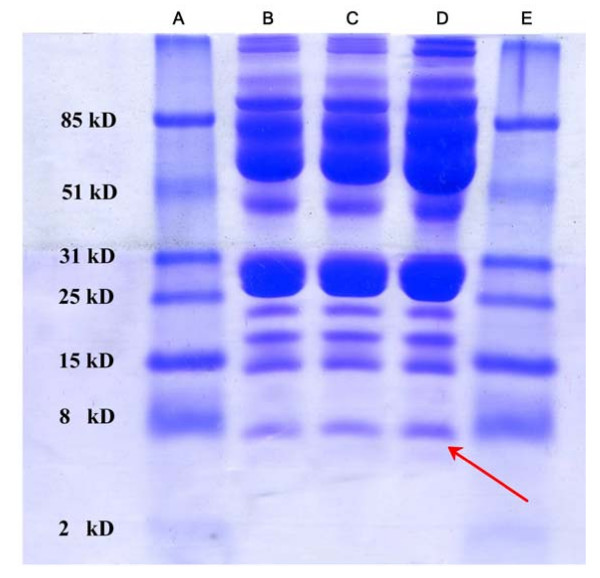
**Isolation of the 7984 m/z peak**. Lane B, C and D: samples; Lane A, and E: molecular marker proteins. Arrow indicates the band of 7984 m/z protein.

To isolate the proteins of interest and to determine candidate protein identities, 6 serum samples from HCC patients containing high SELDI intensity of 7789 M/z and 7984 M/z were selected as potential candidate proteins for isolation by Tricine-SDS-PAGE. Fig. 3 shows a picture of the Tricine-SDS-PAGE gel separating two fractions of proteins with approximate mass between 2.5 km/z and 11 k m/z. The band with 8 m/z mobility that stained more intensively in the gel was excised and trypsinised (Fig. 3).

**Figure 4:**
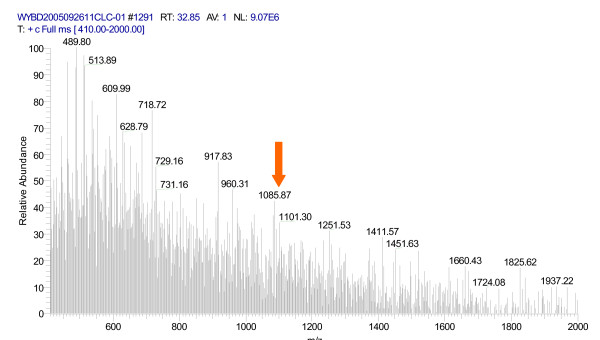
**Peptide mass fingerprinting spectra of the 7984 m/z after trypsin digestion**. The arrow indicates the peptide (1 101.29 m/z) used for subsequent MS/MS analysis.

**Figure 5:**
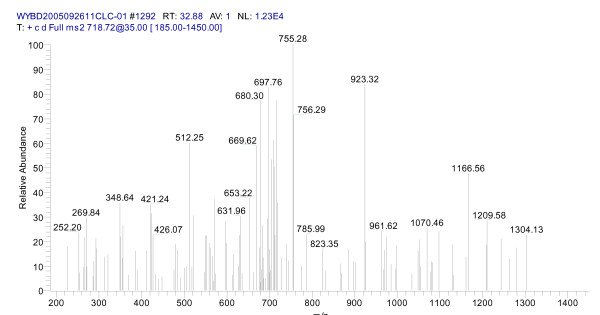
Mass fingerprinting of the 1101.29 m/z peptide (MS/MS analysis).

After trypsin digestion of the 8 k m/z gel band, digested peptides were measured by LC-MS/MS. 79 peptides, including 45 unique peptides, were identified. Fig. 4 and Fig. 5 show the peptide mass fingerprinting of the 7984 m/z band after trypsin digestion. Peptide mass lists derived from spectra with high S/N ratio were submitted to SWISS-PROT/TrAEMBL for database matching. By database search, we found that the sequence of the 7984.14 m/z protein (pI>4) matched with the neutrophil-activating peptide MW = 8 019.36 m/z, pI = 8.43), a protein known for its high expression in liver tumor tissues.

### 2.5 Immunohistochemistry of NAP-2

**Figure 6:**
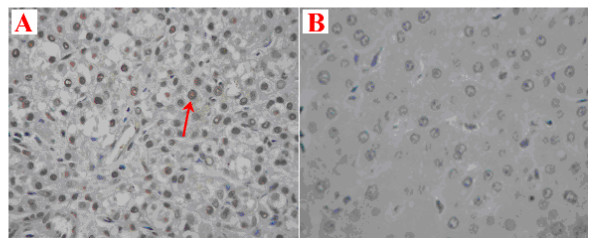
**Immunohistochemical staining of NAP-2 antibody in HCC tissues (A) and adjacent liver tissues (B)**. The positive signals for NAP-2 were observed in brown (6A, red arrow). No positive signal of NAP-2 was detected in adjacent normal liver tissues (6B).

NAP-2 expression in liver tumor tissues and adjacent liver tissues was analyzed by immunohistochemistry using specific NAP-2 antibodies. Interestingly, strong brown staining signals were found in HCC tissues (Fig. 6A), while no NAP-2 staining was found in adjacent liver tissues (Fig. 6B).

## 3. Discussion

In this study, we generated serum protein mass spectra from hepatitis B-related HCC patients and controls. Based on the serum proteomic profiles, we constructed a classifier that accurately distinguishes HCC from controls. Validation of this proteomic classifier in a separate independent testing set showed high accuracy for discriminating HCC cases from controls. One of the proteins (7984 m/z) in the proteomic signature was identified as neutrophil-activating peptide 2 (NAP-2), which was further confirmed by IHC as a specific biomarker of hepatitis B-related HCC.

A major strength of this study is the use of a two-step workflow for proteomic biomarker screening. Several reports have determined that class prediction results should be corroborated in independent sample sets [20-22]. To translate from proteomic peaks to proteomic signatures for discriminating HCC patients from controls, we used BPS to select the best proteomic peaks for construction of classification tree [23]. The classifier separated patients with HCC from healthy controls with a sensitivity of 100% and a specificity 93.94%. To confirm our findings, we subsequently applied our trained classifier to a second, independent test dataset, which was obtained by a rigorous standardized protocol similar to that of training dataset but included a greater proportion of HCC patients. In this new dataset, a classifier trained on the spectra obtained in the first phase of the study discriminated HCC cases from controls with a sensitivity of 100% and specificity of 96.97%. This percentage of correct classification was much higher than the currently accepted biomarkers, such as AFP (46% sensitivity and 89% specificity) [24]. Only one in 48 total HCC samples was misclassified. The fact that the diagnostic performance of the training classifiers survived rigorous testing in testing dataset strengthens the conclusions of the study in the training dataset. Another strength of this study is the utilization of comprehensive proteomic techniques including protein profiling, Tricine-SDS-PAGE and HPLC-MS/MS for detection, identification, and IHC for characterization of NAP-2 as a biomarker in HCC.

Among the proteins identified by MS, one of the top three up-regulated proteins was characterized to be the neutrophil-activating peptide 2 (NAP-2). NAP-2 is a major form of CXCL7, a member of chemokine family involving in regulating immunity, angiogenesis, stem cell trafficking, and mediating organ-specific metastases of cancers [26]. Recent studies using SELDI-based serum proteome profiling, in combination with immunoassays and Western blot analysis, have identified CXCL7 as a marker of advanced myelodysplastic syndromes (MDS), a hematologic stem cell malignancies in elderly patients [13,25]. CXCL7 is translated as a propeptide, then cleaved to several smaller forms each reported to have specific functions. The shortest form, called NAP-2, is structurally related to NAP-1/IL-8, which was shown to be highly expressed in human hepatocellular carcinoma [26] and its serum levels were correlated with clinicopathological features and prognosis of HCC [27]. NAP-2 is a potent activator and attractant for human neutrophils in vitro and in vivo [28]. The expression of NAP-2 mRNA was very low in normal liver tissues but extremely high in liver tumor tissues [29]. Interestingly, no expression of NAP-2 was observed in tissues of other type of tumors, suggesting that NAP-2 probably to be a specific biomarker of liver cancer.

Previous studies using either 2D-gel electrophresis or SELDI-MS in HCC have provided evidence that proteomic biomarkers can be used to discriminate HCC from healthy controls. Reported protein biomarkers included ferritin light chain [30], vitronectin [17], apolipoprotein E, chloride intracellular channel 1 [14], liver aldolase, tropomyosin β-chain, ketohexokinase, enoyl-CoA hydratase, albumin, amooothelin, arginase-1 [31], complement C3a [13], and brian derived neurotrophic factor (BDNF) [32]. Differences between previous studies and this present study might result from the applied methods (comprehensive strategy *vs *single approach), available samples (e.g. serum *vs *tissue), sample preparation (e.g. fractionation), different protein chip (CM10, IMAC-Zn, IMAC-Cu, WCX2), patient or control characteristics, and data analysis with different algorithms.

## 4. Conclusion

In summary, we have identified a set of protein peaks that could discriminate HCC from healthy controls. From the protein peaks specific to HCC disease, we identified and characterized neutrophil-activating peptide-2 as a potential proteomic biomarker of HCC. Further studies with larger sample sizes will be needed to verify this specific protein marker and to address its efficacy, especially with regard to discriminating histologic types of HCC and disease stages. Nevertheless, our study demonstrates a rational approach for identifying HCC biomarkers that could be used for detection and monitoring HCC by proteomic techniques.

## 5. Materials and methods

### 5.1 Patients and samples

With informed consent obtained from each participant, serum samples were collected by the Department of Surgery at the First Affiliated Hospital of Guangxi Medical University, China. Patients with HCC were diagnosed according to standard criteria put forth by the Chinese Society of Liver Cancer [33]. The cancer group consisted of 48 patients with HCC. All HCC cases were histologically confirmed, positive for hepatitis B antigen, and negative for anti-hepatitis C. The controls were 33 healthy volunteers without liver neoplasia, alcoholic cirrhosis, hepatitis B or hepatitis C infection, recruited from routine health examination at the same hospital. The mean age was 46.0 years (ranges from 26 to 75 years) for HCC patients and 41.8 years (ranges from 23 to 60 years) for healthy controls. The characteristics of all subjects are shown in Table 5. Sera of HCC patients was collected before any treatment and randomly divided into two groups: training group and testing group. All blood samples were colleted in the morning before breakfast. Two milliliters of whole blood were obtained and stored at 4°C for one hour and then centrifuged for 10 minutes at 3000 r/min. All serum samples were stored at -80°C before SELDI ProteinChip analysis.

**Table 5:** Characteristics of the subjects

	HCC patients (N = 48)	Controls (N = 33)
Age (years)	46.0 ± 11.8	41.8 ± 10.8
Male/Female	39/9	24/9
Hepatitis B infection (%)	100% (0/48)	0% (0/33)
Hepatitis C infection (%)	0% (0/48)	0% (0/33)

### 5.2 SELDI-TOF-MS analysis of serum protein profiles

Protein profiling of serum samples was performed using the eight-spot format WCX2 (weak cationic exchange) ProteinChip Arrays (Ciphergen Biosystems, Fremont, CA, USA). Frozen serum samples were thawed and spun at 10,000 rpm for 5 min at 4°C. Twenty μl of U9 buffer was added to 10 μl aliquots of each serum sample and placed on ice for 30 min before adding 360 μl WCX-2 buffer. Arrays were prepared as follows: each array was pre-equilibrated 2 × 5 min in 200 μl WCX-2 buffer on a horizontal shaker (MSI Minishaker) before sample addition. The sample supernatant was added and incubated for 1 hr on the shaker. After incubation, the sample was removed, and each spot was washed with 200 μl WCX-2 buffer for 2 × 5 min with agitation. After washing, the array was carefully separated from the bioprocessor and washed briefly with deionized water. 0.5 μl sinapinic acid (SPA) was deposited on the array spots and allowed to air dry.

The ProteinChip Arrays were read by surface-enhanced laser desorption/ionization time-of flight (SELDI-TOF-MS) mass spectrometry (ProteinChip PBS II reader, Ciphergen). This was calibrated using NP20 chips that had been bound with all-in-one standard proteins to set up the parameters. The optimal detection parameter of mass/charge size range was set between 2000 and 10000 M/Z with a maximum of 50000 M/Z. The laser intensity was set at 175 and detector sensitivity was set at 5. An average value of 130 spots was presented for each sample. All samples were detected with the same parameters. All the raw data was normalized with the ProteinChip Software version 3.1 (homogenization of the total ion strength and M/Z). The M/Z sample peaks with more than 2000 M/Z were normalized with biomarker wizard of ProteinChip Software version 3.1 for noise filtering. The first threshold for noise filtering was set at 5, and the second was set at 2. The minimum threshold for clustering was set at10%. Spectrum analysis was performed using the Biomarker Patterns Software.

### 5.3 Bioinformatics and biostatistics

Patients with HCC were split into a training set and a testing set. The training sample set consisted of 33 HCC patients and 33 healthy controls. The protein profiling spectra obtained from the serum samples were normalized using total ion current normalization from Ciphergen's ProteinChip Software version 3.1. Peak labeling was performed by the Biomarker Wizard feature of this software. The intensities of selected peaks were then transferred to Biomarker Pattern Software (BPS) as a 'root node'. On the basis of peak intensity, a threshold was determined by BPS to classify the root node into two child nodes. If the peak intensity of a blind sample was lower than or equal to the threshold, this peak would be labeled as left-side child node. Peak intensities higher than the threshold would be marked as right-side child node. After multiple rounds of decision-making, BPS pooled all labeled samples into a terminal node, where samples were divided as cancer group or control group using the classification tree. Classification of the training set was made to yield the least classification error.

The testing set consisted of serum samples from 48 patients with HCC and 33 control individuals. Using the classification model generated from training dataset, BPS evaluated all of the protein peak intensities for each sample in the testing dataset. It then discriminated HCC and control samples according to their proteomic profile characteristics. Model sensitivity was defined as the probability of predicting HCC cases, while specificity was defined as the probability of predicting healthy controls. A positive predictivity value was defined as the probability of HCC if a test result was positive.

### 5.4 Biomarker purification and identification

Pooled serum samples (n = 6) from HCC patients with high SELDI intensities at 7789 and 7984 m/z were selected. These samples were diluted with 9 M urea, 10 mM Tris/HCI (Ph 7.4) and applied to AKTA Purifier T-900 column system [14]. After sample purification, albumin and immunoglobulin were removed from the serum by 3 GA and then by Protein A. The rest of the fractions were loaded onto a Tricine-SDS-PAGE gel according to the methods of Fountoulakis and Schagger [34,35], using ETTAN II (Amersham Pharmacia) gel electrophoresis system.

Electrophoresis was run at 20 mA for 3 hr. Gels were then stained with Coomassie Brillian Blue. The bands corresponding to the 8000 m/z markers were excised and then destained with two washes of 50 μl deionised water, followed with 50 μl ACN/50 mM/L NH_4_HCO_3 _(1:1, v/v), and dried in a SpeedVac concentrator. The dried gel slices were rehydrated with 10 mM DTT followed by 50 mM IAM (45 min at room temperature in the dark). After several washes with 25 mM NH_4_HCO_3 _and 100% CAN, 20 mg/L-solution of trypsin was added to the gel slices and digestion was allowed to proceed at 37°C for 12 hr.

The trypsin digested sample was loaded onto a C18 reversed-phase column (5 mm × 250 μm, PepMapC18, LC Packings, Amsterdam, The Netherlands), and the peptides were separated by electros pray ionization (ESI, Bruker Esquire 3000, Bruker Daltonik, Bremen, Germany). Proteins were identified by an automated searching algorithm against the SWISS-Protand NCBI protein database.

### 5.5 Immunohistochemistry (IHC)

HCC tissues and adjacent liver tissues (control) were processed according to standard approaches [36]. The anti-NAP-2 serum (1:1600, Immunechem Pharmaceuticals INC, Canada) was applied to both HCC and control slides and incubated in a moist chamber at 4°C overnight. 0.01 ml PBS was used as the negative control in all experiments. Slides cut in parallel to the IHC-treated sections were stained by HE for better identification of the different tissue areas. To avoid interindividual bias of IHC staining differentiations, all slides were determined by an experienced pathologist.

## Competing interests

The author(s) declare that they have no competing interests.

## Authors' contributions

HM initials designed the study and was responsible for laboratory studies, data analysis and revision of the manuscript. QJ initials carried out the SELDI-TOF experiments and drafted the manuscript. RZ initials designed the study and was responsible for critical assessment and revision of the manuscript. XW, MR and ZJ initials carried out all the biomarker purification and identification. QW initials participated in data analysis. YH initials participated in SELDI-TOF experiments and data analysis. ZZ initials participated in the design of the study and helped to draft the manuscript. All authors read and approved the final manuscript.
